# First Measurements of Mixed Floral Traits Influencing *Anacardium occidentale* (Anacardeacae) Attractiveness to Bees in Côte d'Ivoire: Conservation and Agricultural Implications

**DOI:** 10.1002/ece3.72749

**Published:** 2026-01-02

**Authors:** Dolourou Silué, Nicodénin A. Soro, Lombart M. M. Kouakou, Seydou Tiho, Souleymane Konate, Wouter Dekoninck

**Affiliations:** ^1^ Research Ecology Center of Nangui Abrogoua University of Côte d'Ivoire Abidjan Côte d'Ivoire; ^2^ UFR Agroforestry Jean Lorougnon Guédé University of Côte d'Ivoire Daloa Côte d'Ivoire; ^3^ UFR Natural Sciences Nangui Abrogoua University Abidjan Côte d'Ivoire; ^4^ Royal Belgian Institute of Natural Sciences, OD Taxonomy and Phylogeny Brussels Belgium

**Keywords:** agronomic performances, cashew floral traits related to bee' visitations, contents in sugars and amino‐acids, density of flowers, quantity of pollens and nectars, sub‐Saharan Africa

## Abstract

In Sub‐Saharan Africa, cashew plants face challenges in suitable pollination and good agronomic performances. These challenges can largely be attributed to the ability of cashew floral traits in pollinator attraction. However, especially in Côte d'Ivoire, little is known about the roles of morphology and density of cashew flowers and floral rewards in attracting bee species. Likewise, the relationships between plants' attractiveness, number of pollinator visits, and fruiting rate are rarely the focus of study. Therefore, we contrasted in 3 Ivoirian regions two categories of cashew seeing the bees' foraging preference toward their flowers: trees with high foraging intensity versus trees with low activity (respectively called preferred versus non‐preferred plants). Our aim was to know whether the floral traits varied among these categories of plants, and whether this variation might affect bees' foraging intensity and the yield. Results showed that the two categories of cashew were significantly different in density of flowers, quantity of pollens and nectars, and their contents in sugars and amino‐acids in the pollens and nectars, and showed that these floral traits were strongly involved in bee pollinators recruitment (Wilks = 0.002384, df = 1, *p* < 0.0001). These floral traits also significantly increased the bees' visitation networks from 11 to 38 species and their interactions from 984 to 8271 visits, and agronomic performances from 10.63% ± 6.65% to 50.15% ± 5.34%. Floral traits related to bee visitations, if well‐investigated, may be used to identify high‐yielding cashew plants and preserve pollinators.

## Graphical abstract

This paper will contribute to include/use the bees' floral preference (floral traits related to bee' visitations) to find and validate genetic markers that would enable breeding high‐yielding cashew plants and preserving pollinators.

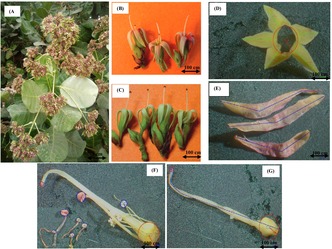

## 1 Introduction

All over the world, both wild and cultivated plants face challenges in suitable pollination and good agronomic performances (Heard et al. 1990; Bhattacharya 2004; O. M. Aliyu 2007). Recent works have demonstrated that bees' pollinators make foraging decisions and preferences toward the flowers based on numerous floral traits like: the nectar and pollen rewards, and associated visual and olfactory cues (Bauer Austin et al. 2017; Prasifka et al. 2018; Glasser et al. 2023). These pollinators may have both innate and learned floral preferences in response to variable resource availability (Bauer Austin et al. 2017; Quintana‐Rodríguez et al. 2018; Glasser et al. 2023). For example, in America, Mallinger and Prasifka (2016) obtained that the bumble bees and honey bees have innate preferences for yellow, blue, and blue‐green flowers including their scent and appearance, corresponding to visual and olfactory cues. Similarly, these pollinators can learn to associate these floral traits with the quantity and quality of nectar and pollen per flower, corresponding to the expected reward and calories available (Bauer Austin et al. 2017; Page et al. 2021). When the expected reward is pollen, these pollinators can visually estimate the pollen quantity per flower and select only the flowers or inflorescences with more pollen rather than flower display size on plants' canopy (Lázaro and Santamaría 2016). Conversely, when the volume of nectar is the expected reward, only the plants containing both larger floral displays and the highest quantities of nectar per flower will be selected by bees' visitors (Lázaro and Santamaría 2016). Furthermore, solitary bees' foraging preferences were associated with higher concentrations of sugars (sucrose, fructose, and glucose) and amino acids in the pollen and nectar rewards (Courcelles et al. 2013; Prasifka et al. 2018). In America, for example, a concentration of 2–6 mM from proline (amino acid) in the floral nectar or pollen seems to increase these pollinators' preference (Quintana‐Rodríguez et al. 2018). The volatile compounds (phenylacetaldehyde) are also attractants to solitary bees' pollinators in Europa (Courcelles et al. 2013). Hence, larger floral displays or inflorescences with more flowers and a high quantity of nectar and pollen usually increase pollinators' visitation, and greater visitation can augment pollen receipt and seed set for plants (LoPresti et al. 2018).

In America and Europa, the importance of floral traits for plant–pollinators interactions has been well studied for both (wild and cultivated) plants and incorporated in breeding programs related to many cultivars like: *Glycine max* (soybean), *Vaccinium corymbosum* (blueberry), *Helianthus annuus* (sunflowers), *Citrus* spp. (citrus) and *Medicago sativa* (Courcelles et al. 2013; Bauer Austin et al. 2017; Prasifka et al. 2018; Mazer et al. 2019). Unfortunately, in Sub‐Saharan Africa, little is known about the floral traits related to bees' visits to plants (Silué et al. 2021). In Côte d'Ivoire for example, where the national economy is building on cash crops like cocoa, coffee, cotton and cashew, the roles of floral traits (floral display size, nectar and pollen rewards, and associated visual and olfactory cues) in attracting bee species are rarely the focus of study (Silué et al. 2021; Silué 2023). Likewise, the relationships between plants' attractiveness due to these floral traits, the pollinators' visitation and seed set are poorly documented (Silué 2023). However, a greater understanding of the relationship between floral traits and bees' foraging behavior could not only improve crop pollination, but could also inform bee conservation efforts (Silué et al. 2022; Glasser et al. 2023; Soro et al. 2024).

Cashew plant ( *Anacardium occidentale* ) originating from Brazil is became the high‐value economic commodity for smallholders in the north of Côte d'Ivoire, and one of most important of cash crop due to its weight in the national economy (IBPGR 1986; Ricau 2019; Silué 2023). Indeed, one variety of cashew (Jumbo) was introduced in 1960s, for the ecological restauration of terrestrial habitats (FIRCA 2018; Ricau 2019). From 1960 to 2018, this variety was propagated with empirical methods by the smallholders using heterogeneous seeds in 20 Ivoirian regions out of 31 existing (CCA 2016). Furthermore, the nuts form these cashew plants has become a second most export crop after cocoa, and one of main monetary income source for smallholders (CCA 2016; FIRCA 2018). Côte d'Ivoire has also become first worldwide producer and exporter of raw cashew nuts with 25% of the global production and 50% of the world's supply (FIRCA 2018; Ricau 2019). Unfortunately, Ivoirian cashew plant faces challenges in suitable pollination and good agronomic performances (Silué et al. 2022; Heard et al. 1990; O. M. Aliyu 2007). These challenges if not properly managed, strongly contributes to the negative impacts on populations' livelihood, food security and income (CCA 2016). Simultaneously, this plant has an excellent reproductive system for determining the specific floral traits that are more attracted and retained the bees' pollinator, corresponding to the probability to increase the pollen transfer and agronomic performance of plants (Wunnachit 1991; Wunnachit et al. 1992; Masawe and Kapinga 2013; Silué 2023). Indeed, cashew plant depend on the cross‐pollination for its seed set and the honey bees ( *Apis mellifera* L.) is its main pollinators (Freitas and Paxton 1998; Freitas et al. 2002; Silué et al. 2021). More recently, two categories cashew plants, seeing the bees' foraging preference toward their flowers were recorded: (i) trees with high foraging activity, called preferred cashew and (ii) trees with low activity, namely non‐preferred. The flowers of the preferred cashew trees were visited 5 times more and they attracted 3 times more bee foragers as compared to non‐preferred (Silué et al. 2021, 2022). In order to provide more insight into this mutualistic interaction between cashew plant and bees' pollinators, and examine the relationships between floral traits, bees' foraging preference and yield, we contrasted these two categories cashew (preferred versus non‐preferred). Our aim was to test whether the floral traits varied among cashew plants from seeds and never grafted, and whether this variation could be affected bees' foraging activity and the yield. We hypothesis that, the quality of cashew floral traits which are likely involved in bees' recruitment can be used as good indicators to identify high yielding cashew plants. To our knowledge, few data have linked the cashew floral traits to bees' foraging activity and yields, as good strategy to enhance productivity and reduce floral resources depletion for pollinators (Wunnachit 1991; Wunnachit et al. 1992; Masawe and Kapinga 2013). This study addresses some of the unanswered questions: 1/what are the key floral traits that attract and retain bees' pollinator on cashew flowers? We predicated that cashew plants that are more visited by bee offer high quality of nectar and pollen including good accessibility to these rewards and good visual which are probably interconnected with olfactory cues. 2/What are the effects of these floral traits on the bees' foraging intensity and cashew yields? We expected that the quality floral traits enhance directly the bees' visit and consequently pollen transfer: the probability to have good agronomic performance of plants.

## 2 Materials and Methods

### 2.1 Description of Cashew Material

*Anacardium occidentale* (Anacardiaceae) is native to north eastern Brazil (IBPGR 1986). Its vegetative structure shows heights ranging from 5 to 20 m with a diameter of the crown fluctuating from 12 to 20 m and a sprawling broad‐leafed ever green (IBPGR 1986). Its root system can grow to 10 m deep or more in soils, with many lateral roots (IBPGR 1986). The cashew plant is well adapted to poor soils and dry sandy locations, but grows best in well‐drained places with a pH of 4.5 to 6.5 (Chipojola et al. 2009; O. M. Aliyu 2007). Its favorable temperatures oscillate from 15°C to 35°C with rainfall of 600 to 2000 mm. The reproductive structure of the cashew plant also shows panicles with hundreds of flowers divided into two groups (male and hermaphrodite) and three phases of flower opening (early male, an intermediate mixed, and a final male phase) with a sex ratio varying from 4% to 17% of hermaphrodite flowers (Sreenivas et al. 2016). This andromonoecious species also shows both flowers (male and hermaphrodite) with white petals on the first day of opening and changing to pink after 3 to 4 days (Wunnachit 1991; Masawe and Kapinga 2013). Male flowers have one large protruding stamen and five to nine small inserted ones (Wunnachit et al. 1992). Hermaphrodite flowers are similar to male flowers except that they also possess a functional pistil, consisting of a stigma, style, and single ovule ovary (Wunnachit et al. 1992). The stigma is usually held higher than the anther in hermaphrodite flowers. Hence, four types of pollen from both stamens (large and small) and two groups of flowers (hermaphrodite and male) were recognized in the inflorescences (Heard et al. 1990; Wunnachit et al. 1992). Cashew plants are grown for their edible kernel within their nuts (akene), swollen peduncle: apple' (hypocarp), and its shell liquid for industry. The color of its apples varies from red to yellow among the trees, and its nuts are of an ever gray color (IBPGR 1986; Bhattacharya 2004).

Study cashews were previously described based on historical information on the plants' origin (seed origin) and their agronomic performances including the bees' activity in the orchards as provided by the growers (Silué et al. 2021, 2022; Silué 2023). It consisted of 60 cashew plants divided into two categories: cashew (preferred versus non‐preferred) seeing the bees' foraging preference toward their flowers. These cashew plants were also described as the variety of Jumbo that was introduced in the 1960s and propagated with heterogeneous seedlings and never‐grafted (CNRA 2015).

### 2.2 Study Area and Sampling Design

**FIGURE 1:**
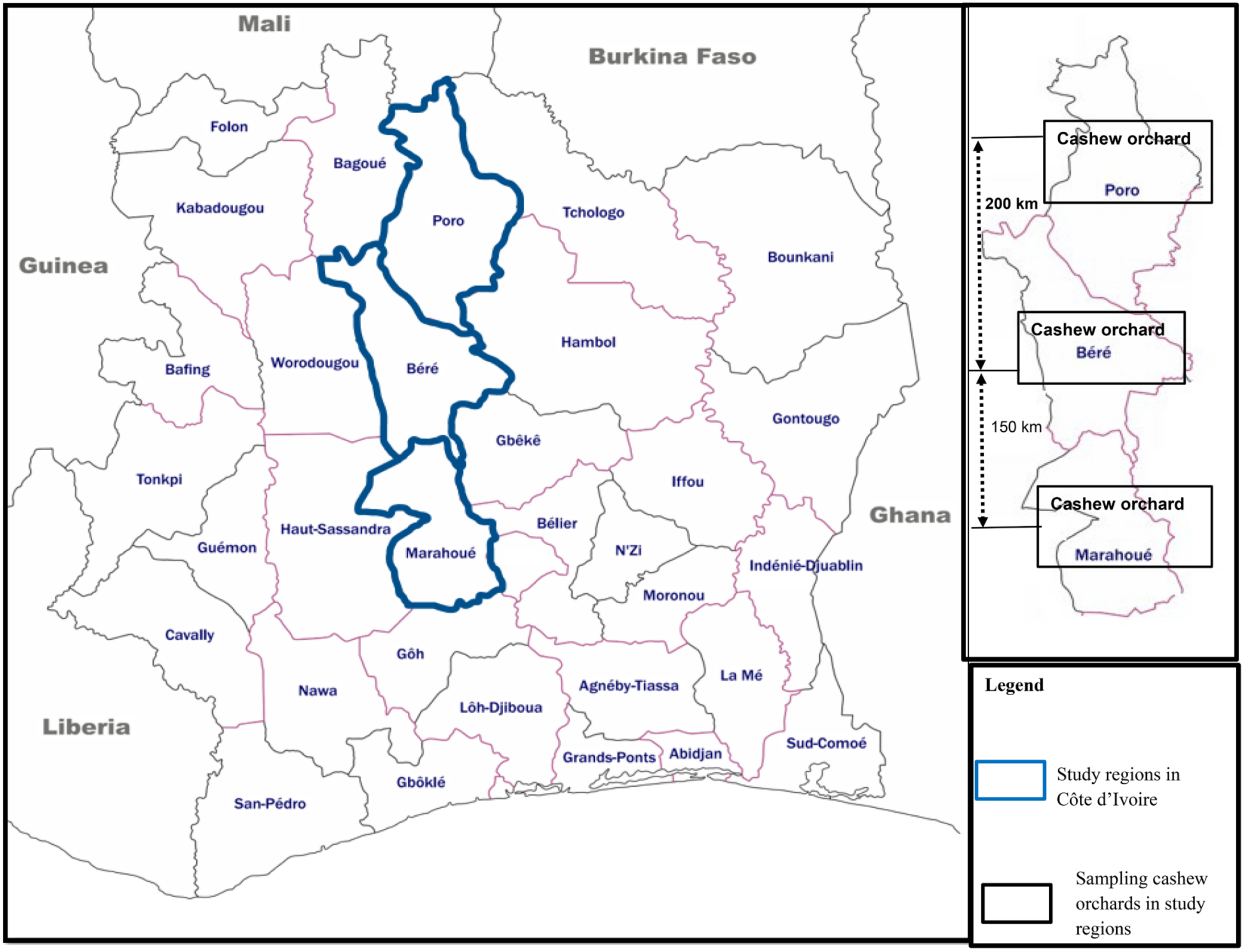
Map showing the location of (C) the sampling sites, (B) study regions, and (A) Côte d'Ivoire in Africa.

The study was conducted in 3 cashew producing regions: Poro, Béré, and Marahoué (Figure 1). The natural vegetation within these regions was dominated by savannahs and island semi‐deciduous forests (Sangare et al. 2009; Goujon et al. 1973). The climate varied from November to April (dry season) and May to October (rainy season) with average annual rainfall of 1000 to 1300 mm (Sodexam 2023). The main economic activity is linked to the cashew crop and the collection of non‐timber forest products, benefiting from favorable climatic conditions.

The sampling design was established following a previous study of Silué (2023), in which the bees' floral preference (preferred and non‐preferred plants) was experimentally detected in each selected region, while the age of sampling cashews was provided by the orchards' owner during an interview (Silué et al. 2022, 2021). Hence, the sampling design concerned one orchard of 10 ha with the two categories cashew: 10 preferred versus 10 non‐preferred, corresponding to a total of 20 mature cashew plants (average age of 20 years per tree) that were considered in each selected region. According to Silué et al. (2021), this selected orchard, including their cashew trees, was recognized as materials from heterogeneous seedling (non‐grafted plants), without beekeeping, and sampling plants were situated in the middle of orchards, at least 4 to 6 km from the natural habitats. The establishment of this sampling design was due to the necessity to avoid the potential effects of non‐study factors that may influence the flowers' visitors such as: the proximity of small and large natural forest fragments, beekeeping, the type of cashew plants (from seedling or grafted plants), and their age. Samples were collected from December 2021 to March 2022 during the bloom period, on a total of 160 inflorescences distributed in 40 individuals for each one of 4 main branches per tree. Data were collected in these 4 branches around the canopy of each tree, especially marked along the four cardinal directions (North, South, East, and West).

### 2.3 Data Collection

#### 2.3.1 Sampling of Floral Traits

Several authors recognized that bee pollinators make foraging decisions and preferences toward the flowers based on their accessibility to good quality rewards (nectar and pollens), which can provide them with necessary food sources, including suitable calories for flying and larvae metabolism (Wunnachit et al. 1992; Bauer Austin et al. 2017; Prasifka et al. 2018; Glasser et al. 2023). Therefore, for each cashew plant, we measured the data related to cashew floral traits as follows: (i) morphology and density of hermaphrodite and male flowers including their inserted stamens per inflorescence, and (ii) average quantities of pollen and nectar and their contents in amino‐acids and sugars. Before data collection, the flowering phenology was recorded on 8 young inflorescences (with non‐opening flowers) to determine: (1) the time of flowers opening by three daily observations and (2) pollen availability by placing a smooth piece of tissue paper on the anthers after flowers opened in order to observe the pollens that have adhered (indication of anthers dehiscence).

**FIGURE 2:**
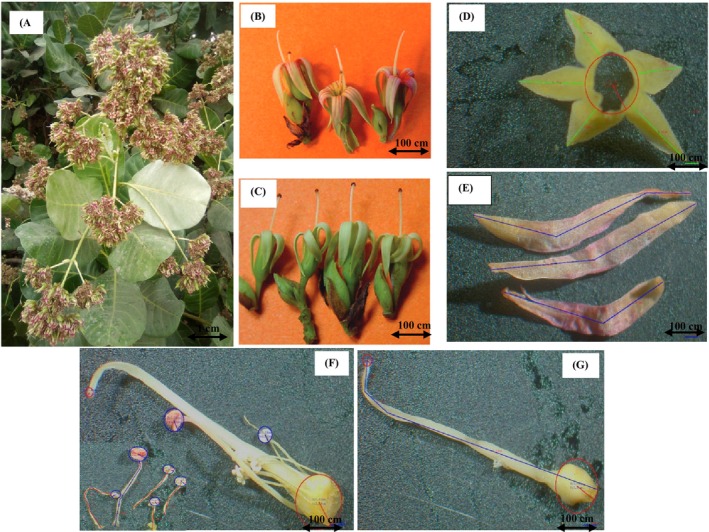
Stalls showcasing (A) density of flowers per inflorescence, (B) hermaphrodite flowers, (C) male flowers and dissected flowers with morpho‐metrics measurement process under microscope of (D) receptacle, (E) petal, (F) stamens and (G) style.

In the field, the density of flowers per inflorescence (expressed in individuals/inflorescence) were determined per cashew plant, considering one sample of 8 inflorescences divided in 2 individuals according to the four cardinal directions. At the same time, the nectar volume per flower (expressed in μL/flower) was measured between 07:00 and 12:00 h on 20 fresh‐opening flowers per tree (10 male and 10 hermaphrodite) using 1 μL microcapillary tube. Total volumes of nectar per inflorescence (expressed in μL/inflorescence) were estimated by multiplying the average quantity of nectar per flower by the density of flowers per inflorescence. Conversely, 20 flowers per tree (10 male and 10 hermaphrodite) were collected and conveyed to the laboratory to record both width and length of corolla tube and inserted stamens including the diameter of their anthers, corresponding to the morphology of flowers (Figure 2). The measurements were done according to Wunnachit et al. (1992) with modifications, regarding the dissecting process of flowers for effective collection of data on each floral organ. Hence, both tubes (floral and corolla) were separated. The perimeter at the base of corolla tube was taken as its width (expressed in mm), while the height of tube or distance from the base to the opening, was considered as its length (expressed in mm). Furthermore, the floral tube was gently dissected to separate the pistil and stamen types (Figure 2). Stamen types (large and small) were separately counted and measured to collect their morphometric data like: (i) individual perimeters of dissected anther types (expressed in mm), considered as individual width of stamen types, (ii) individual height of stamens or distance from the anther to the inserted point with ovary (expressed in mm), taken as its length, and (iii) diameters of dissected anthers using its perimeter divided by *pi*. These data were collected under microscope stereo motic SMZ‐171 equipped with 0.01 mm precision stainless steel digital caliper (Traceable Products, Webster, Texas, USA). Rationale behind these measurements was the necessity to determine whether the floral morphology limits access of pollinators to food sources (nectar and pollens), and whether average quantity from these foods varied among the study cashews including the flowers types.

Similarly, anthers from 20 flowers per tree (10 male and 10 hermaphrodite) were gently forced open to allow counting of the pollen per stamen types (large and small) and flower types. Two total of pollens per flower types per inflorescence in relation with four sub‐total of stamen types (two large and two small for the two flower types) were also estimated by multiplying the number of pollens per stamen types by two densities: inserted stamens per flower and flowers per inflorescence. Prior to this calculation, pollens grains were also counted on biology molecular laboratory under electronic microscope in three replications for each sample, and respectively expressed in individuals/large or small stamen, individuals/flower and individuals/inflorescence. Obtained samples (pollens and nectar) were also examined for amino‐acids and sugars contents using high‐performance liquid chromatography apparatus ((Gilson Inc., Middelton, WI, USA) equipped with RI K‐2300 refractometric detector (Knauer, Berlin, Germany)) following Wunnachit et al. (1992). Before that, samples of nectar form the flowers types on one hand, and samples of pollens from small and large stamens per flower on other hand were weighed, grounded in 90% ethanol and centrifuged. Obtained supernatant were also analyzed for sugar and amino‐acids contents. Sugars were identified and their relative masses quantified using a waters Associates SugarPak column (30 cm × 6.5 mm), 410 refractive index detector, and WISP 710B automatic injector. The samples injection volume was 100 μL with a running time of 31 min at a constant temperature of 90°C. Quantities of each sugar in fresh weight of pollen and nectar (respectively expressed in μg/mg and μg/mL) were determined in comparison to standards of fructose, glucose, and sucrose. Similarly, amino acids analysis was conducted using a waters PICO TAG 30 cm column, a waters model 481 spectrophotometer detector, and a 710B WISP TM auto injector and column heater. Amino acids were derivatized with phenylisothiocyanate as described by Wunnachit et al. (1992) and separated by reverse‐phase‐chromatography. The samples injection volume was 25 μL with a running time of 73 min at a constant temperature of 38°C. Amino acids in pollen and nectar (respectively expressed in nmol/mg and nmol/mL) were identified by comparing retention times in the samples against standards, and quantified by comparing the peak areas. Rationale behind these measurements was the necessity to determine whether the calories or energies provided by pollens and nectar to bee' pollinators, varied among the study cashews including the flowers types.

#### 2.3.2 Sampling of Bee' Pollinators

Bees' foraging intensity from is recognized as measurable effects induced by the floral traits on insect pollinators (Bauer Austin et al. 2017; Prasifka et al. 2018). Therefore, for each study cashew plant, we assessed the floral visitors following Vaissière et al. (2011) with modifications, regarding the rate of data collection. Around the canopy of each study tree, two observers walked to capture flowers visitors on the 4 main branches per tree at the following times: 7 am; 9 am; 11 am; 1 pm; 3 pm; 5 pm, during 3 consecutive sunny days (20 min per tree and sampling hour, including 5 min per branch). In each sampling time, insects capturing was simultaneously done in two opposite parts of canopy: (i) North and South in 5 min and (ii) East and west in 5 min. Capturing was done with entomological nets, only the insects they could see on the flowers of the branch immediately in front of them. Later, bees' specimens were identified in the entomological laboratory of Research Station in Lamto using the determination keys of Michener (2007); Eardley et al. (2010), Olympus SZ61 binocular loupe and the reference collection of bees. During this identification process, recorded data per sampling hour were: (1) bees' foraging intensity or interactions, expressed in visits/min/h and (1) species richness, expressed species/h, for the 160 inflorescences per tree. In addition, these data were pooled to have the total visitation of bees (expressed in visits/tree/day) and total species richness (expressed in species/tree) for future statistical analyses. Rationale behind these measurements was the necessity to determine whether the community of bee' pollinators, effectively attracted by the floral resources, varied among the study cashews.

#### 2.3.3 Sampling of Cashew Yields

Agronomic performance of cashew is recognized as measurable effects of pollens transferring, particularly carried out by bees (Wunnachit et al. 1992; Masawe and Kapinga 2013). Hence, for each study cashew plant, we assessed three parameters related to agronomic performance like: (i) fruiting rate, (ii) weight (g) of nuts, and (iii) their kernel outturn, as described by Masawe (2006) and Masawe and Kapinga (2013). The fruiting rate (expressed in percentage of observed fruits per tree) was measured on 160 mature inflorescences per tree, by individual counting of hermaphrodite flowers that have been pollinated by bees. The quality of cashew nuts or useful kernel per tree (expressed in lbs. per tree) was assessed on 50 raw nuts/tree following A.C.I (2010). Prior to this determination, the cashew nuts were carefully cut using dissecting forceps in order to weigh separately kernels, pellucids, shells and calculate the useful kernels (100% of good kernel +50% of dotted kernel by insects +50% premature kernels). The rationale behind these measurements was the necessity to determine whether the yields varied among the study cashews.

### 2.4 Data Analyses

#### 2.4.1 Statistical Determination of Key Floral Attributes That Retaining Bee' Pollinators

To determine the key floral attributes that attract and retain bee' pollinators, the two categories cashew (preferred and non‐preferred) were contrasted and the relative difference among the data were interpreted as key floral traits that are involved in bee' recruitments. These comparisons cover both global and specific aspects of assessed floral traits including bees' recruitment and cashew yields. Multidimensional scaling (MDS) analysis applied with vegan R‐package (version 4.3.2; R Core Team 2023) was first done to compare the global aspects of cashew floral traits like: (i) density of total flowers and inserted stamens, and (ii) quantities of total pollens and nectar and their contents in amino‐acids and sugars. Based on the relative difference among these data, a MANOVA test using stats R‐package was carried out for depth analyses of the specific floral traits which are included in these global aspects of floral attributes. Analyses of specific floral traits correspond to the multiple comparisons of: (1) individual density of flowers and inserted stamens types, (2) individual measurements of width and length of stamen' types and corolla of flower' types, and (3) average quantities of both nectar and pollen of flower' types and their contents in amino‐acids and sugars. Post hoc test from Tukey was finally used to separate these individual variables. These statistical analyses were used due to normal distribution and homoscedasticity of data that was respectively verified with Shapiro–Wilk and Bartlett' tests.

#### 2.4.2 Statistical Determination of Relationship Between the Key Floral Attributes, Bees' Community and Yields

To examine effects of cashew floral traits on bees' foraging intensity and yields, a Quasi‐Poisson regression model using the pscl R‐package was necessary to assess the association among the following data: (i) density of total flowers and inserted stamens and quantities of total pollens and nectar and their contents in amino‐acids and sugars, (ii) total visitation of bees and species richness and (iii) total fruit counting per inflorescence, fruiting rate, weight of individual nut and usefulness of kernel in the nuts. These statistical analyses were done due to over‐dispersion of quantitative data (Jackman 2015). In addition, the MANOVA test was used to compare the two categories' cashew (preferred and non‐preferred) and relative differences were interpreted as measurable effects from the key floral attributes in enhancing bees' foraging intensity and yields. These multiple comparisons cover the following parameters: (1) total visitation of bees and species richness and (2) total fruits counting per inflorescence, fruiting rate, weight of individual nut and usefulness of kernel in the nuts. ANOVA with Bonferroni correction was finally carried out to separate these individual parameters. These statistical analyses were used due to normal distribution and homoscedasticity of data that was respectively verified with Shapiro–Wilk and Bartlett's tests.

Complementary analyses were also carried out to understand the potential role of cashew plants as agro‐melliferous plants for bees' conservation. These analyses included: (i) building of two pollination networks, resulting in bees' visitation in the flowers of studied cashews (Dormann et al. 2009), and (ii) their key metrics comparison, using linear mixed model with sampling orchards as a random effect, to test the differences among the structures from these plants‐pollinators networks. This statistical analysis was used due to normal distribution and homoscedasticity of data that was respectively verified with Shapiro–Wilk and Bartlett's tests. Assessed metrics include: connectance, nestedness, modularity, specialized networks (H2), interaction evenness, links per species, Shannon index and network size (Dormann et al. 2009). These metrics including plants‐pollinators networks were generated using the R package ‘bipartite'v.2.15, excepted to the network size (M = A + P): one metric which was obtained by accumulating the number of plant (P) and bees (A) species. Based on linear mixed model comparisons, the two networks were also separated, while their metrics were interpreted following Almeida‐Neto et al. (2008) with modifications, regarding their ecological role. For example, high values from three assessed metrics (links per species, network size and Shannon index) were taken as ability of cashew florals to attract high diversity of bee’ pollinators. Conversely, low values from four assessed metrics like: connectance, modularity, nestedness, interaction evenness and complementary specialization (H2′), were understood as ability of cashew florals to establish a stable relationship with this high diversity of pollinators.

## 3 Results

### 3.1 Floral Traits Explaining Bees' Foraging Preference

**FIGURE 3:**
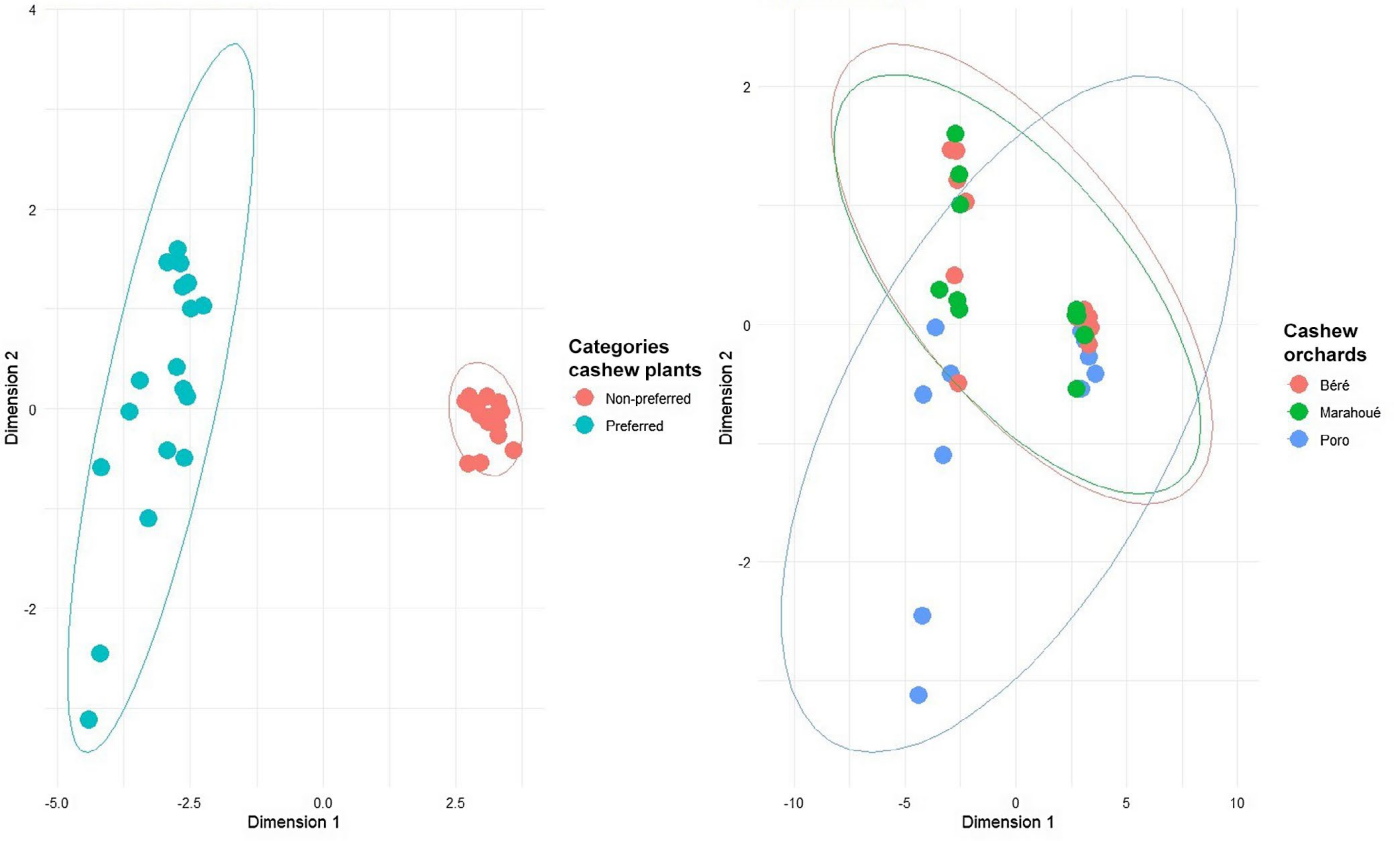
Multidimensional scaling (MDS) analysis of global aspects of floral attributes, cashew yields, and bees' recruitment in relation to sampling plants and study sites. Colors and shapes indicate categories of cashew plants and study sites.

Multidimensional scaling (MDS) analysis for global aspects of floral traits separated the study cashews into two categories (preferred and non‐preferred) and showed overlap between all study sites (Figure 3). Overall results showed that floral traits (density of total flowers and inserted stamens and quantities of total pollens and nectar and their contents in amino‐acids and sugars) were significantly involved in bee' pollinators attraction (Wilks = 0.002384, df = 1, *p* < 0.0001).

Specifically, eight key floral traits form morphology and density of flowers and their inserted stamens were significantly attractant to bees (Wilks = 0.00244, df = 1, *p* < 0.0001). Results showed that the high density of flowers' types recorded on preferred compared to non‐preferred cashews (523 ± 46 male and 34.1 ± 4 hermaphrodite flowers per inflorescence, versus 244.1 ± 47 and 20 ± 3 individuals respectively) were significantly involved in bee' pollinators recruitment (*F*_1,59_ = 362.84, *p* < 0.0001). The high density of inserted stamens in the flowers from preferred compared to non‐preferred cashews (11.6 ± 0.5 individuals per male and 10.6 ± 0.5 individuals per hermaphrodite flowers, versus 8 ± 1 and 7 ± 0.3 respectively) were also significantly involved in bee' pollinators recruitment (*F*_1,59_ = 220.58, *p* < 0.0001). The high values from diameters of both small and large anthers from preferred compared to non‐preferred cashews (0.2 ± 0 for small and 0.4 ± 0 mm for large anthers in both male and hermaphrodite flowers, versus 0.1 ± 0 and 0.3 ± 0 mm respectively) were significantly involved in bee' pollinators recruitment (*F*_1,59_ = 572.72, *p* < 0.0001; Table 1A).

**TABLE 1:** Summary table showing the analysis from (A) morphology and density of flowers including inserted stamens, (B) average quantities of nectar and pollen, (C) sugars' contents in nectar and pollen, and (D, E) amino‐acids' contents in pollen and nectar.

(A) Morphology and density of flowers and inserted stamens														
Categories cashew	Mf*** (*n* = 8)	Hf*** (*n* = 8)	N.stM*** (*n* = 10)	N.stH*** (*n* = 10)	l.sstM (*n* = 10)	l.lstM (*n* = 10)	l.sstH (*n* = 10)	l.lstH (*n* = 10)	D.satH*** (*n* = 10)	D.latH*** (*n* = 10)	D.satM*** (*n* = 10)	D.latM*** (*n* = 10)	D.rM (*n* = 10)	D.rH (*n* = 10)
Preferred (*n* = 30)	523 ± 46	34.1 ± 4	11.6 ± 0.5	10.6 ± 0.5	1.3 ± 0.1	4 ± 0.1	1.3 ± 0.1	4 ± 0	0.2 ± 0	0.4 ± 0	0.2 ± 0	0.4 ± 0	1 ± 0.1	1.1 ± 0.1
Non‐preferred (*n* = 30)	244.1 ± 47	20 ± 3	8 ± 1	7 ± 0.3	1.3 ± 0.1	4 ± 0.1	1.3 ± 0.1	4 ± 0	0.1 ± 0	0.3 ± 0	0.1 ± 0	0.3 ± 0	1 ± 0.1	1 ± 0.2

(B) Average quantities of nectar and pollen										
Categories cashew	Vm* (*n* = 10)	Vh*** (*n* = 10)	VTm*** (*n* = 10)	VTh*** (*n* = 10)	VT*** (*n* = 10)	Categories cashew	GE. pfM*** (*n* = 10)	ge.pfM*** (*n* = 10)	GE.pfH*** (*n* = 10)	ge.pfH* (*n* = 10)
Preferred (*n* = 30)	0.07 ± 0.01	0.17 ± 0.02	36.4 ± 9	5.6 ± 1.2	42 ± 10	Preferred (*n* = 18)	949.1 ± 39	161 ± 10	907 ± 168	151 ± 11
Non‐preferred (*n* = 30)	0.04 ± 0.01	0.07 ± 0.01	10.4 ± 3.5	1.4 ± 0.3	12 ± 3.6	Non‐preferred (*n* = 18)	730 ± 94	124 ± 4	788 ± 62	128.4 ± 10.6

(C) Sugars contents in nectar and pollen														
Categories cashew	EH1. gl*** (*n* = 10)	eH1.gl*** (*n* = 10)	EH1.fr*** (*n* = 10)	eH1.fr*** (*n* = 10)	EM1.gl*** (*n* = 10)	eM1.gl*** (*n* = 10)	EM1.fr*** (*n* = 10)	eM1.fr*** (*n* = 10)	NH.gl*** (*n* = 10)	NH.fr*** (*n* = 10)	NH.Su*** (*n* = 10)	NM.gl*** (*n* = 10)	NM.fr*** (*n* = 10)	NM.Su*** (*n* = 10)
Preferred (*n* = 18)	100 ± 5	51 ± 0.6	123.3 ± 1.6	55.3 ± 0.8	20.2 ± 0.7	15.9 ± 0.5	23.2 ± 0.8	19 ± 0.6	345.5 ± 8	353.7 ± 8	60.13 ± 0.7	332.6 ± 46	336 ± 49	147.6 ± 4.2
Non‐preferred (*n* = 18)	75.8 ± 2.7	40.5 ± 1	117.5 ± 72.5	46.4 ± 0.8	14.6 ± 0.5	14 ± 0.5	20.2 ± 0.7	15.9 ± 0.5	203.5 ± 3	207.1 ± 3.3	34.5 ± 1.4	203 ± 2.5	207.7 ± 2.8	124.3 ± 1.4

(D) Amino‐acids contents in pollen														
Categories cashew	Asp*** (*n* = 10)	Glq*** (*n* = 10)	Ser*** (*n* = 10)	Aspg*** (*n* = 10)	Gly*** (*n* = 10)	Glu*** (*n* = 10)	His*** (*n* = 10)	Threo*** (*n* = 10)	Ala*** (*n* = 10)	Arg*** (*n* = 10)	Pro*** (*n* = 10)	Tyr*** (*n* = 10)	Val*** (*n* = 10)	Met*** (*n* = 10)
Preferred GE (H) (*n* = 18)	7.7 ± 0.5	13 ± 1	21.4 ± 1.5	4.4 ± 0.6	8.6 ± 0.5	24.6 ± 1.4	2 ± 0.2	5.7 ± 0.3	17.6 ± 1	17.6 ± 1	157.3 ± 15	4.3 ± 0.6	20.1 ± 2	0.2 ± 0
Non‐Preferred GE (H) (*n* = 18)	3.4 ± 0.2	5.2 ± 0.7	9.2 ± 0.3	1.3 ± 0.2	3.5 ± 0.2	11.7 ± 0.6	0.3 ± 0	2 ± 0.2	8.5 ± 1	8.5 ± 1	88 ± 3.6	1 ± 0.1	9.3 ± 1.3	0.1 ± 0
Preferred ge (H) (*n* = 18)	11.2 ± 1	14.6 ± 0.5	17.3 ± 0.6	3.6 ± 0.3	8.7 ± 0.3	18.3 ± 1.1	3.1 ± 0.1	3.7 ± 0.26	11.5 ± 1.2	11.2 ± 1.3	30.4 ± 3.7	2 ± 0.2	22.2 ± 0.7	0.3 ± 0.1
Non‐preferred ge (H) (*n* = 18)	4.1 ± 0.2	6.9 ± 0.4	8.7 ± 0.3	1.1 ± 0.1	3.6 ± 0.2	10.1 ± 1	0.2 ± 0	1.1 ± 0.1	5 ± 1	5 ± 1	12.2 ± 1	0.3 ± 0	10.8 ± 0.6	0.1 ± 0
Preferred GE (M) (*n* = 18)	2.1 ± 0.3	3.7 ± 0.7	4.5 ± 0.5	9.5 ± 0.5	1.8 ± 0.1	7 ± 0.4	0.5 ± 0.1	1 ± 0.3	4.6 ± 0.2	4.6 ± 0.2	34 ± 0.7	1.3 ± 0.3	7.1 ± 0.3	0.23 ± 0
Non‐preferred GE (M) (*n* = 18)	0.1 ± 0	0.2 ± 0	1.6 ± 0	3.4 ± 0.5	0.2 ± 0	2.3 ± 0.3	0.1 ± 0	0.1 ± 0	1.4 ± 0.4	1.4 ± 0.4	20.1 ± 2	0.1 ± 0	2.7 ± 0.3	0.1 ± 0
Preferred ge (M) (*n* = 18)	2.1 ± 0.4	3.6 ± 0.7	4.5 ± 0.5	9.2 ± 0.7	1.7 ± 0.3	7.1 ± 0.4	0.5 ± 0.1	1 ± 0.3	4.6 ± 0.3	4.6 ± 0.2	34 ± 0.7	1.3 ± 0.3	7.1 ± 0	0.2 ± 0
Non‐preferred ge (M) (*n* = 18)	0.1 ± 0	0.2 ± 0	1.6 ± 0	3.4 ± 0.5	0.2 ± 0	2.3 ± 0.3	0.1 ± 0	0.1 ± 0	1.4 ± 0.4	1.4 ± 0.4	20.1 ± 2	0.1 ± 0	2.7 ± 0.3	0.1 ± 0

(E) Amino‐acids contents in nectar														
Categories cashew	Asp*** (*n* = 10)	Glq*** (*n* = 10)	Ser*** (*n* = 10)	Aspg*** (*n* = 10)	Gly (*n* = 10)	Glu (*n* = 10)	His (*n* = 10)	Threo*** (*n* = 10)	Ala*** (*n* = 10)	Arg*** (*n* = 10)	Pro*** (*n* = 10)	Tyr (*n* = 10)	Val*** (*n* = 10)	Met*** (*n* = 10)
Preferred NE(H) (*n* = 18)	0.15 ± 0	0.2 ± 0	0.13 ± 0	0.1 ± 0	ND	ND	ND	0.05 ± 0	0.15 ± 0.2	0.15 ± 0.2	0.5 ± 0	ND	0.07 ± 0	0.03 ± 0
Non‐preferred NE(H) (*n* = 18)	0.06 ± 0	0.1 ± 0	0.12 ± 0	0.02 ± 0	ND	ND	ND	0.01 ± 0	0.01 ± 0	0.01 ± 0	0.2 ± 0	ND	0.02 ± 0	0.01 ± 0
Preferred NE(M) (*n* = 18)	0.25 ± 0	0.3 ± 0	0.3 ± 0	0.2 ± 0	ND	ND	ND	0.06 ± 0	0.13 ± 0	0.13 ± 0	0.54 ± 0	ND	0.08 ± 0	0.03 ± 0
Non‐preferred NE(M) (*n* = 18)	0.1 ± 0	0.16 ± 0	0.09 ± 0	0.12 ± 0	ND	ND	ND	0.02 ± 0	0.03 ± 0	0.03 ± 0	0.15 ± 0	ND	0.02 ± 0	0.01 ± 0

*Note:* Mf: density of male flowers (expressed in individuals/flower); Hf: density of hermaphrodite flowers (expressed in individuals/flower); N.stM: density of inserted stamens in male flowers (expressed in individuals/flower); N.stH: density of inserted stamens in hermaphrodite flowers (expressed in individuals/flower); l.sstM: length of small stamens form male flowers (expressed in mm); l.lstM: length of large stamens form male flowers (expressed in mm); l.sstH: length of small stamens form hermaphrodite flowers (expressed in mm); l.lstH length of large stamens form hermaphrodite flowers (expressed in mm); D.satH: width of small anther from hermaphrodite flowers (expressed in mm), D.latH: width of large anther from hermaphrodite flowers (expressed in mm); D.satM width of small anther from male flowers (expressed in mm); D.latM width of large anther from hermaphrodite flowers (expressed in mm); D.rM: width of corolla from male flowers (expressed in mm); D.rH: width of corolla from hermaphrodite flowers (expressed in mm). Vm: Nectar volume per male flower (expressed in μL/flower); Vh: Nectar volume per hermaphrodite flower (expressed in μL/flower); VTm: Total volume of nectar in the male flowers per inflorescence (expressed in μL/inflorescence); VTh: Total volume of nectar in hermaphrodite flowers per inflorescence (expressed in μL/inflorescence); VT: Total volume of nectar in the inflorescences (expressed in μL/inflorescence). GE. pfM: Pollens quantity in large stamen per male flower (expressed in individuals/flower); ge.pfM: Pollens quantity in small stamen per male flower (expressed in individuals/flower); GE.pfH: Pollens quantity in large stamen per hermaphrodite flower (expressed in individuals/flower); ge.pfH: Pollens quantity in small stamen per hermaphrodite flower (expressed in individuals/flower); EH1. Gl: Glucose content in the pollens from large stamens of hermaphrodite flowers (expressed in μg/mg); eH1.gl: Glucose content in the pollens from small stamens of hermaphrodite flowers (expressed in μg/mg); EH1.fr: Fructose content in the pollens from large stamens of hermaphrodite flowers (expressed in μg/mg); eH1.fr: Fructose content in the pollens from small stamens of hermaphrodite flowers (expressed in μg/mg); EM1.gl: Glucose content in the pollens from large stamens of male flowers (expressed in μg/mg); eM1.gl: Glucose content in the pollens from small stamens of male flowers (expressed in μg/mg); EM1.fr: Fructose content in the pollens from large stamens of male flowers (expressed in μg/mg); eM1.fr: Fructose content in the pollens from small stamens of male flowers (expressed in μg/mg); NH.gl: Glucose content in the nectar from hermaphrodite flowers (expressed in μg/mL); NH.fr: Fructose content in the nectar from hermaphrodite flowers (expressed in μg/mL); NH.Su: Sucrose content in the nectar from hermaphrodite flowers (expressed in μg/mL); NM.gl: Glucose content in the nectar from male flowers (expressed in μg/mL); NM.fr: Fructose content in the nectar from male flowers (expressed in μg/mL); NM.Su: Sucrose content in the nectar from male flowers (expressed in μg/mL). Asp: Aspartique content in pollen and nectar (respectively expressed in nmol/mg and nmol/mL); Glq: Glutamique content in pollen and nectar (respectively expressed in nmol/mg and nmol/mL); Ser: Serine content in pollen and nectar (respectively expressed in nmol/mg and nmol/mL); Aspg: Asparagine content in pollen and nectar (respectively expressed in nmol/mg and nmol/mL); Gly: Glycine content in pollen and nectar (respectively expressed in nmol/mg and nmol/mL); Glu: Glutamine content in pollen and nectar (respectively expressed in nmol/mg and nmol/mL); His: Histidine content in pollen and nectar (respectively expressed in nmol/mg and nmol/mL); Threo: Threonine content in pollen and nectar (respectively expressed in nmol/mg and nmol/mL); Al: Alanine content in pollen and nectar (respectively expressed in nmol/mg and nmol/mL); Arg: Arginine content in pollen and nectar (respectively expressed in nmol/mg and nmol/mL); Pro: Proline content in pollen and nectar (respectively expressed in nmol/mg and nmol/mL); Tyr: Tyrosine content in pollen and nectar (respectively expressed in nmol/mg and nmol/mL); Val: Valine content in pollen and nectar (respectively expressed in nmol/mg and nmol/mL); Met: Methionine content in pollen and nectar (respectively expressed in nmol/mg and nmol/mL); ND: not detected. *Note:* Asterisks indicate significant differences among floral traits (ANOVA with Bonferroni correction: *p < 0.05, **p < 0.001, ***p < 0.0001).

In addition, the four key floral traits form the quantities of pollens and nectar and their contents in amino‐acids and sugars were significantly attractant to bees. Results showed that the high quantities of nectar form flowers' types of preferred compare to non‐preferred cashews (42 ± 10 versus 12 ± 3.6 μL of total volume per inflorescence) were significantly involved on bees' pollinators recruitment (*F*_1,59_ = 572.72, *p* < 0.0001). The high quantities of pollens form flowers' types of preferred compare to non‐preferred cashews (949.1 ± 39 individuals for large anther of male, 161 ± 10 individuals for small anther of male and 907 ± 168 individuals for large anther of hermaphrodite and 151 ± 11 individuals small anther of hermaphrodite, versus 730 ± 94 and 124 ± 4 and 788 ± 62 and 128.4 ± 10.6 individuals respectively for this four types of anthers) were also significantly involved on bees' pollinators recruitment (*F*_1,59_ = 66.8; *p* < 0.0001). The high of quantities of sugars (*F*_1,59_ = 627.53, *p* < 0.0001) and amino‐acids (*F*_1,59_ = 3366.85, *p* < 0.0001) form flowers of preferred compare to non‐preferred cashews were significantly involved on bees' pollinators recruitment (Table 1B–E).

Conversely, the two categories cashew (preferred and non‐preferred) did not differ significantly in length values of stamens (1.3 ± 0.1 mm for small and 4 ± 0.1 mm for large in both male and hermaphrodite flowers, versus 1.3 ± 0.1 mm and 4 ± 0 mm respectively) and showed that they were low involved on bee' pollinators recruitment (*F*_1,59_ = 0.18, *p* = 0.84). The two categories of cashews did not differ significantly in diameter values of floral corolla (1 ± 0.1 mm for male and 1.1 ± 0.1 mm for hermaphrodite, versus 1 ± 0.1 and 1 ± 0.2 mm respectively) and also showed that they were low involved on bees' pollinators recruitment (*F*_1,59_ = 0.78, *p* = 0.46; Table 1A).

Furthermore, 14 types of amino‐acids (Aspartique, Glutamique, Serine, Asparagine, Glycine, Glutamine, Histidine, Threonine, Alanine, Arginine, Proline, Tyrosine Tyr, Valine, Methionine) were detected in pollens, while 10 were found in nectar, corresponding to four types of amino‐acids that were not recorded in nectar like: Glycine, Glutamine, Histidine, Tyrosine. Results showed that the quantities of nectar and pollen including their contents in sugars and amino‐acids were significantly higher in hermaphrodite flowers compare to male flowers. Two types sugar (glucose and fructose) were found in pollen whereas three (glucose, fructose and sucrose) were detected in nectar (Table 1D,E).

### 3.2 Effect of Floral Traits on Bees' Foraging Intensity and Cashew Yields

**FIGURE 4:**
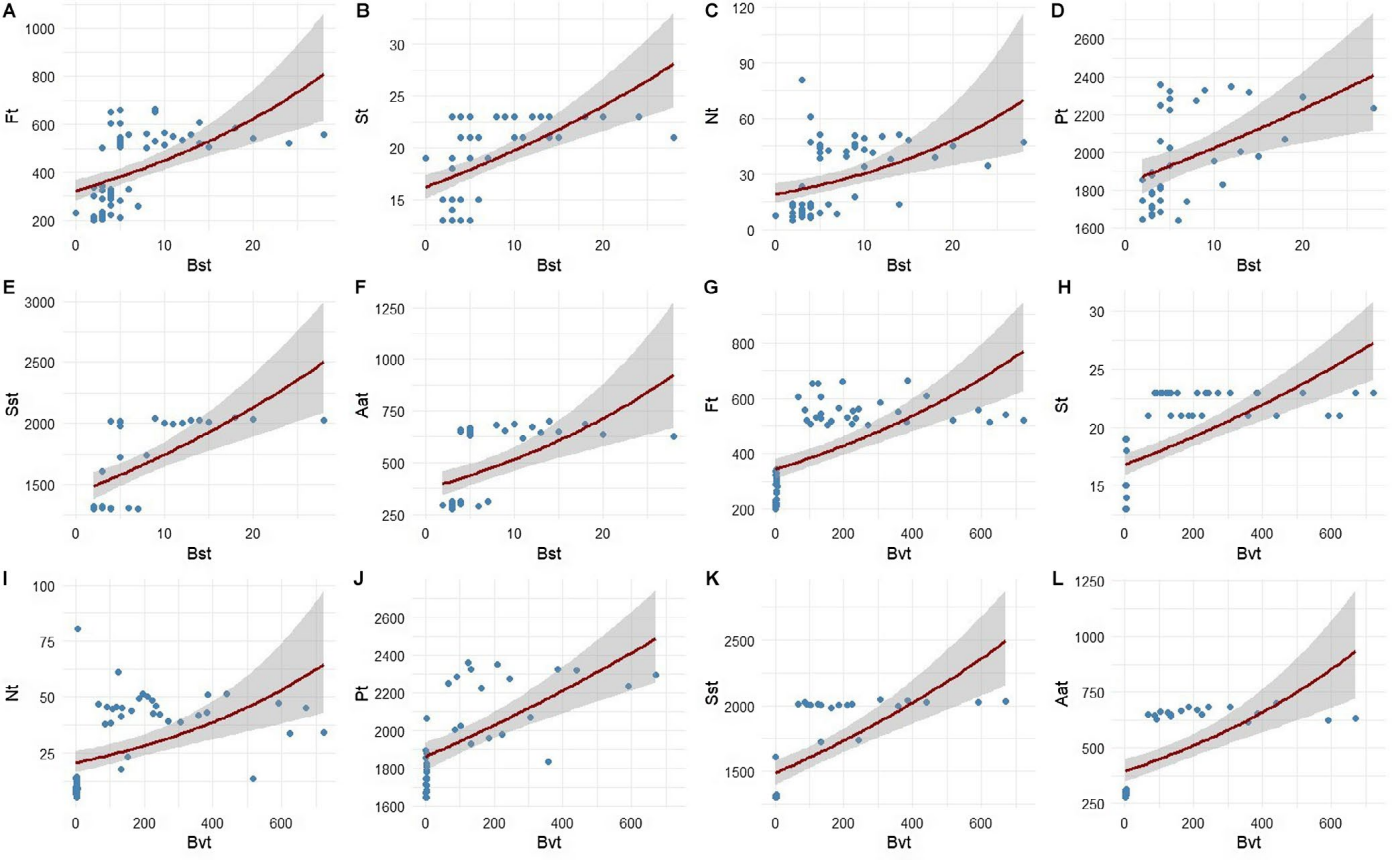
Plots from the Quasi‐Poisson regression model, showing the effect of cashew floral traits on bees' foraging intensity. (A) effect of density of total flowers (Flt) on bees' species richness (Bst); (B) effect of density of total inserted stamens (Stm) on bees' species richness (Bst); (C) effect of total quantity of nectar (Nct) on bees' species richness (Bst); (D) effect of total quantity of pollens (Plt) on bees' species richness (Bst); (E) effect of density of total contents in sugar (SSt) form pollens and nectar on bees' species richness (Bst); (F) effect of density of total contents in amino‐acids (Aat) form pollens and nectar on bees' species richness (Bst); (G) effect of density of total flowers (Flt) on bees' visitation (Bvt); (H) effect of density of total inserted stamens (Stm) on bees' visitation (Bvt); (I) effect of density of total quantity of nectar (Nct) on bees' visitation (Bvt); (J) effect of density of total quantity of pollens (Plt) on bees' visitation (Bvt); (K) effect of density of total contents in sugar (SSt) form pollens and nectar on bees' visitation (Bvt); (L) effect of density of total contents in amino‐acids (Aat) form pollens and nectar on bees' visitation (Bvt). Shaded areas represent the 95% confidence intervals around the predicted values.

Density of flowers and inserted stamens and quantities of both pollens and nectar and their contents in amino‐acids and sugars were positively and strongly correlated with the high intensity of bee visits and species richness (Figure 4 and Table 2). Results of cashews comparison (non‐preferred versus preferred) showed that these floral traits significantly increased (Wilks = 0.002384, df = 1, *p* < 0.0001) bees' species richness from 11 to 38 species and their interactions from 984 to 8271 visits in the flowers.

**TABLE 2:** Summary statistics of Quasi‐Poisson model, showing the cashew floral traits affecting the recruitment of bees and fruits.

	Cashew floral traits	Parameters of cashew yields												Parameters of bees' foraging intensity in cashew flowers					
		Fct			Frt			Nwt			KORt			Bvt			Bst		
		Estimate	*t*	Pr(>|*t*|)	Estimate	*t*	Pr(>|*t*|)	Estimate	*t*	Pr(>|*t*|)	Estimate	*t*	Pr(>|*t*|)	Estimate	*t*	Pr(>|*t*|)	Estimate	*t*	Pr(>|*t*|)
	Intercept	6.8	3.18	0.0024**	4.2	3.01	0.038*	7.33	3.18	0.0025**	0.17	3.5	0.00151**	4.56	2.01	0.034*	4.56	2.01	0.04*
	Flt	0.006	15.8	< 2e‐16***	0.004	11.019	7.54e‐16***	−0.001	−8.544	7.55e‐12***	0.0008	15.3	< 2e‐16***	0.007	6.15	7.44e‐08***	0.003	5.75	3.47e‐07***
	Stm	0.25	13.6	< 2e‐16***	0.19	12.9	< 2e‐16***	−0.05	−5.927	1.8e‐07***	0.034	14.6	< 2e‐16***	0.4	6.07	1.04e‐07***	0.13	5.82	2.62e‐07***
	Nct	0.03	8.3	1.73e‐11***	0.024	7.14	1.7e‐09***	−0.009	−4.9	7.8e‐06***	0.005	9.01	1.26e‐12***	0.03	4.36	5.36e‐05***	0.02	4.2	9.17e‐05***
	Plt	0.003	6.3	3.54e‐07***	0.002	5.97	9.25e‐07***	−0.0007	−4.5	6.13e‐05***	0.0004	7.08	3.46e‐08***	0.004	4.32	0.000128***	0.02	3.4	0.00184**
	SSt	0.003	11.6	2.26e‐13***	0.002	12.06	7.77e‐14***	−0.0007	−6.4	2.59e‐07***	0.0004	12.8	1.29e‐14***	0.005	5.25	8.11e‐06***	0.002	5.5	3.45e‐06***
	Aat	0.006	17.0	< 2e‐16***	0.004	14.111	8.95e‐16***	−0.001	−6.5	1.88e‐07***	0.0008	16.9	< 2e‐16***	0.009	4.49	7.68e‐05***	0.003	4.78	3.24e‐05***

*Note:* Asterisks indicate significant correlation (*p < 0.05, **p < 0.001, ***p < 0.0001).

Abbreviations: Aat, total contents in amino‐acids form pollens and nectar (expressed in nmol/mg and nmol/mL); Bst, total bees' species richness; Bvt, total bees' visitation (visits/min).; Fct, fruits counting (expressed in individuals/inflorescence); Flt, density of total flowers (expressed in individuals/flower); Frt, fruiting rates (expressed in percentage); KORt, nut quality or kernel outtrun ratio (lbs); Nct, total quantity of nectar (expressed in μL/inflorescence); Nwt, nuts weight (g); Plt, total quantity of pollens (expressed in individuals/inflorescence); SSt, total contents in sugars form pollens and nectar (respectively expressed in μg/mg and μg/mL); Stm, density of total inserted stamens (expressed in individuals/flower).

**FIGURE 5:**
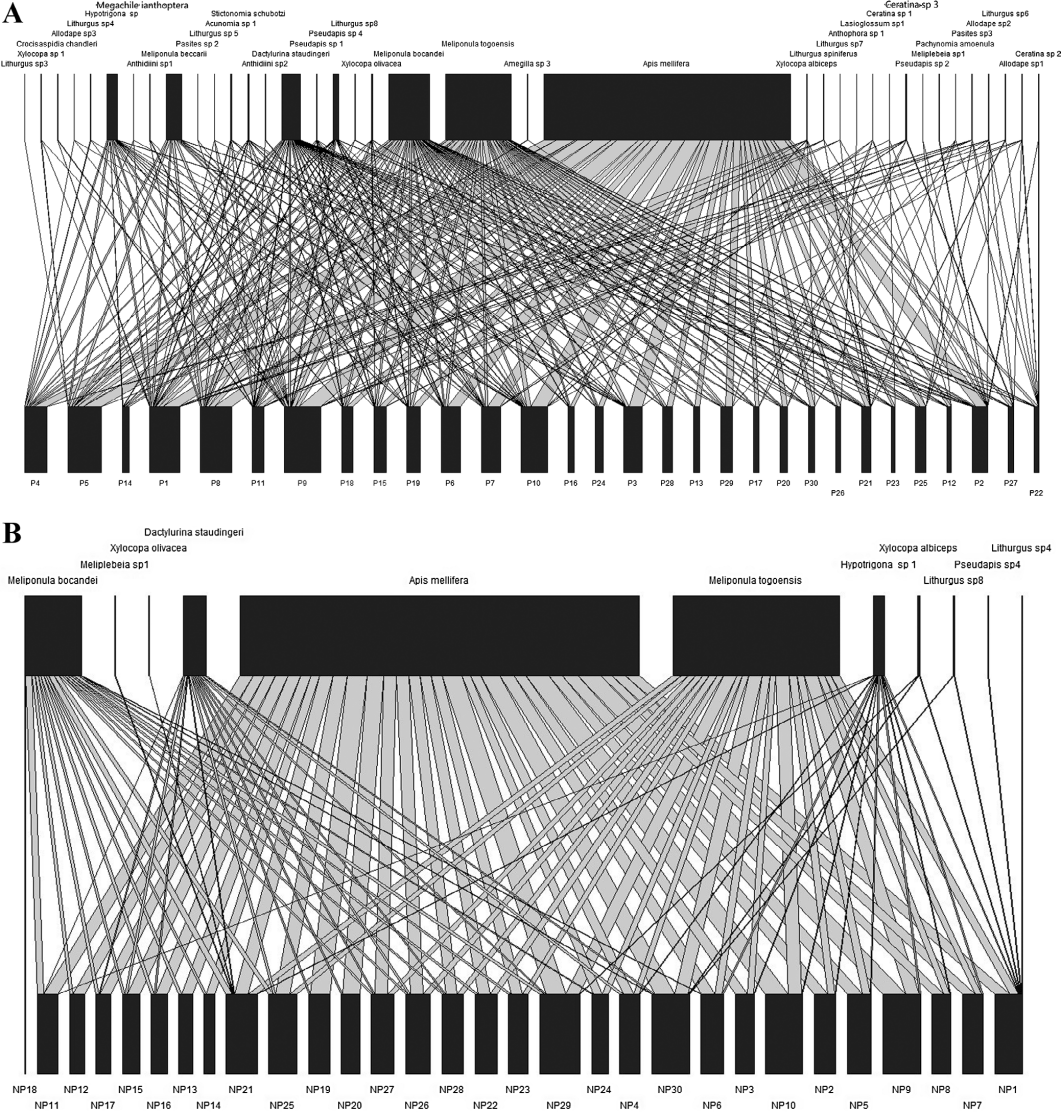
Plant‐pollinator networks for both (A) preferred versus (B) non‐preferred cashews, constructed using the R package ‘bipartite’ v.2.15. Bee pollinators are displayed as rectangles at the top, and the cashew plants' identity (ranged from 1 to 30 for each category) is shown as rectangles at the bottom of each bipartite graph. The width of the rectangles represents the relative frequency of interactions of each species.

Bee' visitations were significantly diversified and more stable (LMM; *Z* = 2.369, *p* = 0.017) in preferred cashews compare to non‐preferred, and showed that the floral resources from preferred plants may play a major role as agro‐melliferous plants for bees' conservation (Table 3). Results showed that the floral traits significantly increased (LMM; *Z* = 2.369, *p* = 0.017), the size of bees' visitation networks from 41 to 68 and number of interactions per bee' species from 2.51 to 4.11 and Shannon index from 4.23 to 4.5 0.1 and nestedness from 33.96 to 37.93. Conversely, the quality of floral traits significantly decreased (LMM; *Z* = 2.369, *p* = 0.017), the insects' connectance from 0.31 to 0.24 and interaction evenness from 0.73 to 0.6 ± 0.1 and network specialization (H2′) from 0.18 to 0.09 and modularity from 0.12 to 0.8 (Figures 4 and 5 and Table 3).

**TABLE 3:** Network' metrics form cashew‐bees interaction including their ecological interpretations.

Categories cashew	Network metrics related to the stability of pollinators visitation					Network metrics related to the diversity of interactions					*p*
	Connectance	Specialization index	Modularity	Nestedness	Interaction evenness	Shannon index	Total interactions	Network size	Bees' species	Links per species	
Preferred (*n* = 1)	0.25	0.08	0.08	38.46	0.64	4.5	8271	68	38	4.13	0.017
Non‐preferred (*n* = 1)	0.31	0.18	0.125	33.96	0.73	4.2	984	41	11	2.5	
Individual comparison of metrics (preferred versus non‐preferred)	Low	Low	Low	High	Low	High	High	High	High	High	—
Ecological interpretation	Bees' visitation was more stable in preferred cashew plants					Bees' visitation was more structured and diversified in preferred cashew plants					

Bees' visitation networks also showed that honeybee and the two stingless bees (respectively *Apis mellifera, Meliponula bocandei* and *Meliponula togoensis* ) were the most abundant flowers visitors of cashew plants (Figure 5).

**FIGURE 6:**
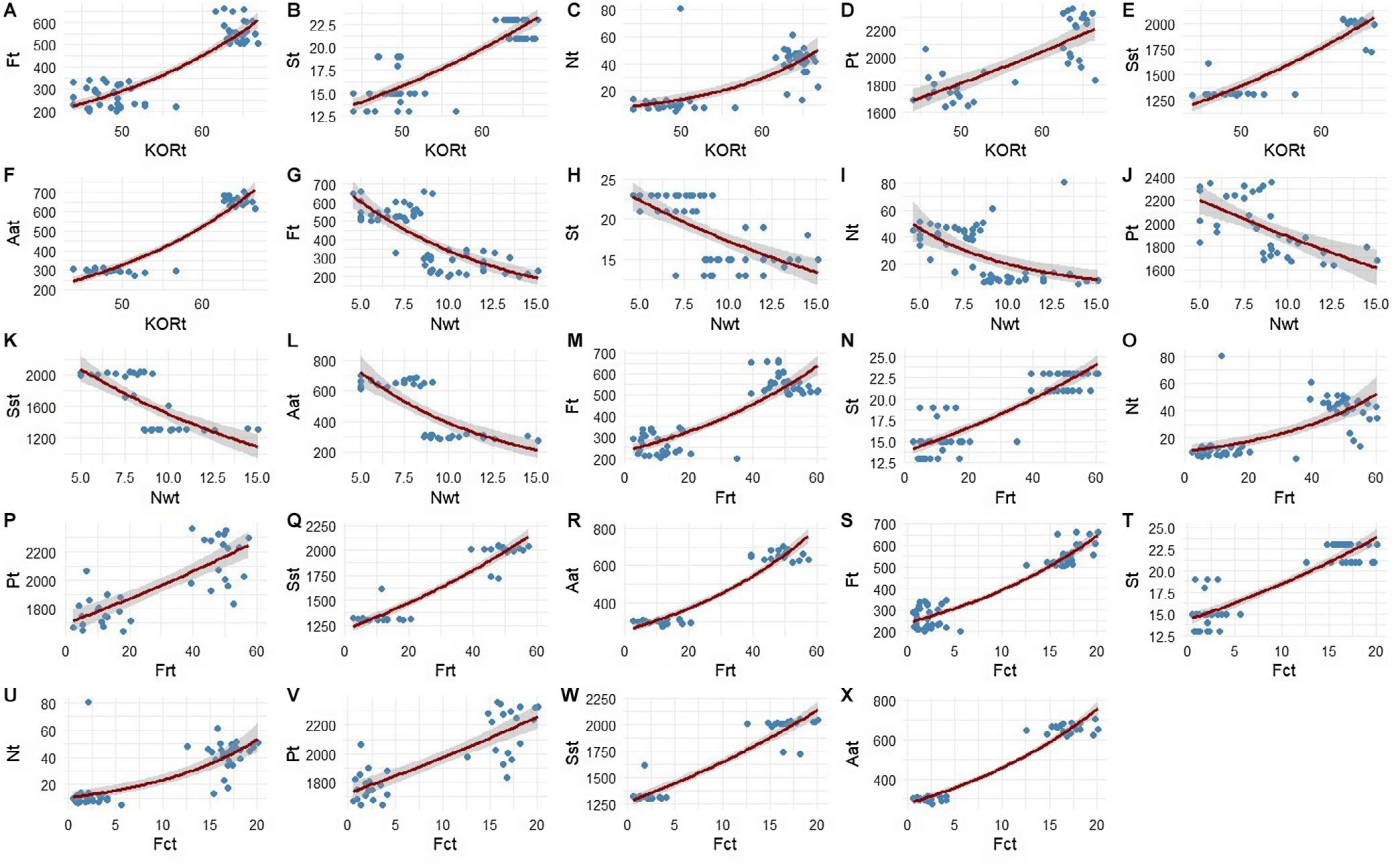
Plots from the Quasi‐Poisson regression model, showing the effect of cashew floral traits on yields. (A) effect of density of total flowers (Flt) on fruiting rates (Frt); (B) effect of density of total inserted stamens (Stm) on fruiting rates (Frt); (C) effect of total quantity of nectar (Nct) on fruiting rates (Frt); (D) effect of total quantity of pollens (Plt) on fruiting rates (Frt); (E) effect of total contents in sugar (SSt) form pollens and nectar on fruiting rates (Frt); (F) effect of total contents in amino‐acids (Aat) form pollens and nectar on fruiting rates (Frt); (G) effect of density of total flowers (Flt) on fruits counting (Fct); (H) effect of total inserted stamens (Stm) on fruits counting (Fct); (I) effect of total quantity of nectar (Nct) on fruits counting (Fct); (J) effect of total quantity of pollens (Plt) on fruits counting (Fct); (K) effect of total contents in sugar (SSt) form pollens and nectar on fruits counting (Fct); (L) effect of total contents in amino‐acids (Aat) form pollens and nectar on fruits counting (Fct); (M) effect of density of total flowers (Flt) on nuts weight (Nwt); (N) effect of total inserted stamens (Stm) on nuts weight (Nwt); (O) effect of total quantity of nectar (Nct) on nuts weight (Nwt); (P) effect of total quantity of pollens (Plt) on nuts weight (Nwt); (Q) effect of total contents in sugar (SSt) form pollens and nectar on nuts weight (Nwt); (R) effect of total contents in amino‐acids (Aat) form pollens and nectar on nuts weight (Nwt); (S) effect of density of total flowers (Flt) on nut quality or kernel outtrun ratio (KORt); (T) effect of density of total inserted stamens (Stm) on nut quality or kernel outtrun ratio (KORt); (U) effect of total quantity of nectar (Nct) on nut quality or kernel outtrun ratio (KORt); (V) effect of total quantity of pollens (Plt) on nut quality or kernel outtrun ratio (KORt); (W) effect of total contents in sugar (SSt) form pollens and nectar on nut quality or kernel outtrun ratio (KORt); (X) effect of total contents in amino‐acids (Aat) form pollens and nectar on nut quality or kernel outtrun ratio (KORt). Shaded areas represent the 95% confidence intervals around the predicted values.

**FIGURE 7:**
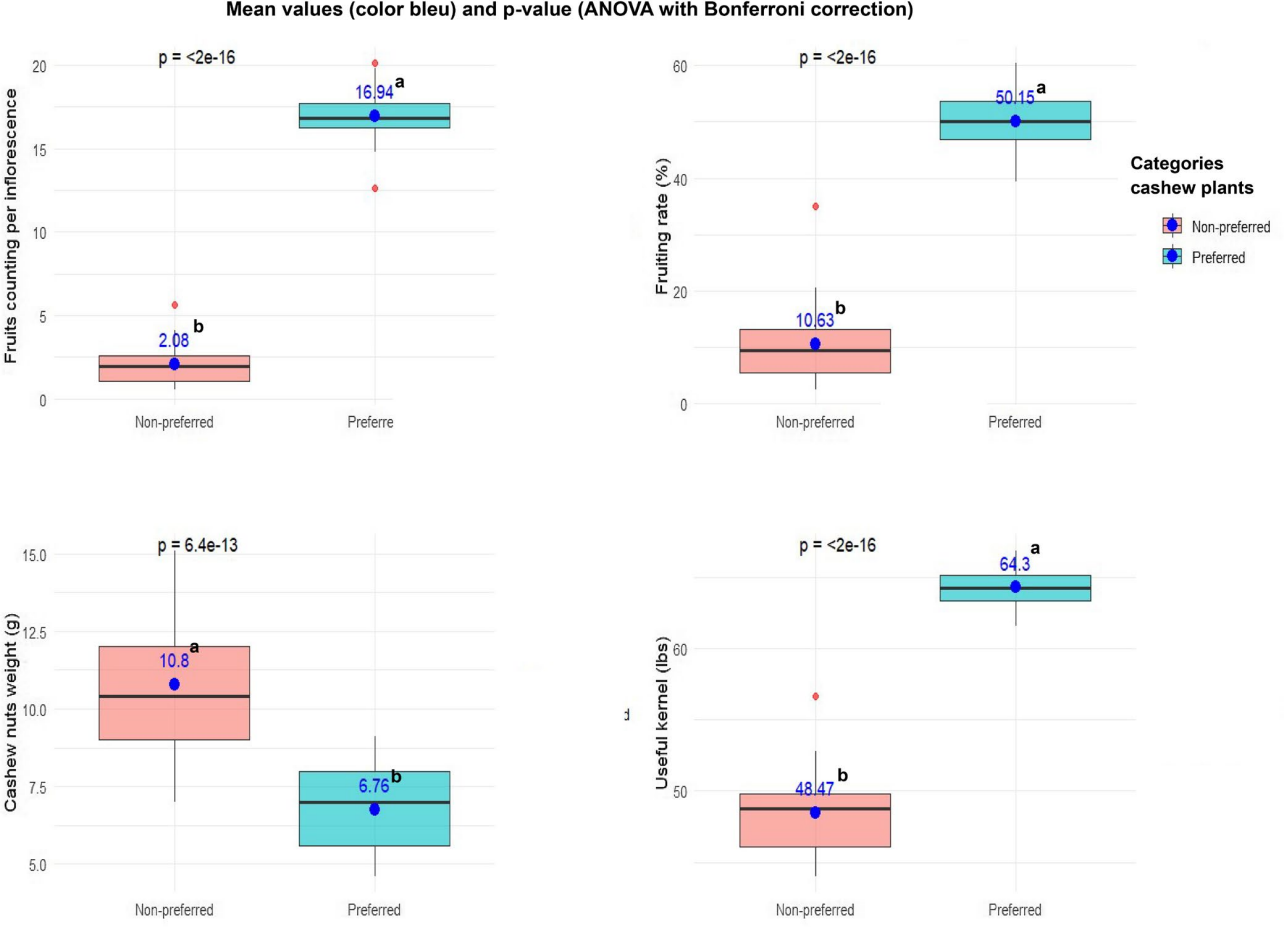
Boxplots showing agronomic performances of cashews' categories. Letter (a and b) indicating significant (*p* < 0.05) differences between the cashews (preferred and non‐preferred).

In addition, the density of flowers and inserted stamens and quantities of pollens and nectar and their high contents in amino‐acids and sugars were positively and strongly correlated with fruiting rate and cashew nuts' quality, and negatively correlated with the individual weight of nuts (Figure 6). Results of cashews comparison (non‐preferred versus preferred) showed that these floral traits significantly increased (*F*_1,59_ = 66.8; *p* < 0.001) the fruits counting per inflorescence from 2.1 ± 1.2 to 16.9 ± 1.55 individuals, and fruiting rate from 10.63% ± 6.65% to 50.15% ± 5.34% including cashew nuts quality or useful kernel from 48.47 ± 2.9 to 64.3 ± 1.24 lbs. Conversely, they significantly decreased (*F*_1,59_ = 66.8; *p* < 0.001) the individual weight of cashew nuts from 10.79 ± 2 to 6.75 ± 1.34 g per nut (Figures 6 and 7).

## 4 Discussion

Our research has yielded comprehensive data which indicated a significant difference between floral traits from the two categories cashew (preferred and non‐preferred) and facilitated our understanding regarding the bees' foraging preference and its strong implication in enhancing cashew agronomic performances, through pollen transferring. Overall results showed that the density of flowers and floral rewards (amounts of both pollens and nectar including their contents in sugars and amino‐acids) are the key floral traits that were significantly involved in bees' attractiveness and yields' improvement. Our findings align with the previous studies which demonstrated that attraction of pollinators is based on floral display size, pollens and nectar secretion and their chemical components (Knudsen and Tollsten 1993; Faegri and Van der Pijl 2013). For example, it was reported that many fruit and vegetable crops with strong dependence on pollinators, including blueberry (Jabłonski et al. 1984), watermelon (Wolf et al. 1999), raspberries and blackberries (Schmidt et al. 2015), and zucchini (Roldán‐Serrano and Guerra‐Sanz 2005) show positive associations between bee visits and nectar volume or total sugar per flower, and these traits may help address needs of agriculture and pollinators.

### 4.1 Density of Flowers and Floral Rewards and Bees' Foraging Preference

Results showed that the preferred cashews have significant density of flowers and they received significant bee visits compared to non‐preferred. The density of flowers per inflorescence was then consistently involved in bee attraction. This floral trait may be associated with visual cues and probably interconnected with olfactory cues, which are recognized to strongly and positively influence the bees' visitation rates (Bauer Austin et al. 2017; Prasifka et al. 2018; Glasser et al. 2023). Its apparition (this trait) much reflects the variations among the cashew plants within the Ivoirian orchards and probably due to non‐adapted agricultural practices such as the use of heterogeneous seedlings for cashew propagation since its introduction in the 1960s in Côte d'Ivoire (Masawe and Kapinga 2013).

Results also showed that the preferred cashews have significant quantities of nectar and pollens compare to non‐preferred and indicated that the quantity of these two floral rewards is strongly involved in bees' attractiveness. Excepted the amounts of pollen which is directly linked to density of inserted stamens and the diameter of anthers, both floral rewards (nectar and pollen) are directly associated to density of flowers, rather than the corolla‐tube width and length. These results may indicate the interactions/combinations among specific floral traits for bees' pollinator attraction (Schiestl and Johnson 2013; Dar et al. 2017; Kariyat et al. 2021) and demonstrated that floral morphology, especially the corolla‐tubes don't limit bees' accessibility to rewards as the pollen and nectar. Subsequently, both long and sort‐tongued bees' pollinator (respectively *Apis mellifera* and *Pseudapis interstitinervis* ) were collected on cashew flowers. We found interestingly that these floral characters, if well investigated, may contribute to the preservation of bees' diversity in cashew orchards (O. M. Aliyu 2004; Campbell et al. 2012). The evolution of genetic material could explain these: (i) correlations among the floral traits and (ii) high dynamic of amounts from rewards (nectar and pollens) secretion between cashew plants (Bhattacharya 2004; O. M. Aliyu 2007; Chipojola et al. 2009).

Results showed that the preferred cashews have significant contents in sugars and amino‐acids from pollens and nectar compared to non‐preferred and indicated that the chemical components of these floral rewards are strongly involved in bees' attractiveness. The geographic origin of cashew trees (native to the north eastern Brazil and introduced in Côte d'Ivoire) and their adaptation to the new environmental conditions (temperature, humidity and soil) in Côte d'Ivoire could probably induce the apparition of these floral traits that affect bees' preference due to contents in sugars and amino‐acids from pollens and nectar (Wunnachit et al. 1992; Masawe and Kapinga 2013). Specifically, the contents in sugars were significantly higher in hermaphrodite flowers compared to male flowers. These results could explain why the foraging activity of bee' pollinators is higher during the hermaphrodite flowering phases compared to the male phase and could be due to the fitness capacity which contributes to the reproductive success of cashew plants (Masawe et al. 2015). Sreenivas et al. (2016) indicate that a phase was considered as “male phase” as long as male flowers continued to be more in number as compared to hermaphrodite flowers and vice versa.

### 4.2 Floral Preference and Bees' Foraging Intensity

Results showed that the density of flowers and inserted stamens and quantities of pollens and nectar and their contents in amino‐acids and sugars were positively and strongly correlated with the intensity of bee visits and species richness. The quality of genetic material of preferred plants could induce their high rewarding capacity, especially during the dry season, where there are few flowers for bees' pollinators in the North of Côte d'Ivoire, and could explain these results (Wunnachit et al. 1992; Masawe and Kapinga 2013; Silué 2023). In addition, the high nutritional quality of rewards in terms of calories could affect bee pollinators' attraction, and therefore explain these results (Quintana‐Rodríguez et al. 2018).

Specifically, the floral resources from preferred cashews may play a major role as agro‐melliferous plants for bees' conservation. These results could probably explain the high occurrences of pollinators community in the flowers of preferred cashews. The higher values of network metrics related to the diversity of interactions in the flowers from this category plants could explain these results, and probably revealed the strong link between floral quality and the existence of multiple interactions of pollinators (Bauer Austin et al. 2017).

Results showed that honeybee and the two stingless bees (respectively *Apis mellifera, Meliponula bocandei* and *Meliponula togoensis* ) were the most abundant flower visitors of cashew plants. The social structure of these two Apidae (a single colony provides thousands of individual visitors to flowers), affects their great demand for pollen and nectar for the larvae, adults, and beehives (Eardley 2004; Eardley et al. 2010) and consequently explains these results. The flowers' morphology is probably more attractive to these two Apidae, and therefore explains their high activities (Mazi et al. 2020). These bee species are generalists in terms of food and nesting resources and seem to benefit in: (i) large monocultural crops, where a great pollination effort is required; (ii) ecosystems where populations of natural pollinators are reduced due to lack of adequate habitat and the use of pesticides; and (iii) seasonal crops that precede the annual activity of pollinator insects. Furthermore, we also think that the two stingless bees *Meliponula bocandei* and *Meliponula togoensis* are likely to provide complementary pollination services to cashew plants, especially when the apicultural is absent from the orchards. These stingless bees have multiple characteristics that make them less likely to encounter the reproductive structures of the flower such as their small size, strong preference for nectar, and behavior of perching on the petals rather than on the anthers and pistils (Michener 2007).

### 4.3 Bees' Foraging Preference and Cashew Agronomic Performance

Results showed that the density of flowers and inserted stamens and quantities of pollens and nectar and their contents in amino‐acids and sugars were positively and strongly correlated with high fruiting rate and cashew nuts' quality, and negatively correlated with the high individual weight of nuts. Ours finding showed that preferred cashews have both high fruit set and quality of nuts compare to non‐preferred. Several interacting mechanisms likely drove this shift: (i) the evolution of genetic material of preferred cashew plants due to human breeding might positively affect the attractiveness of flowers and therefore explain their high agronomic performances, (ii) the high recruitments of foliage and twigs on canopies (IBPGR 1986; Roe 1994) might also positively affect the activity of *Oecophylla longinoda* for the trees' protection and consequently explain these results (Masawe 2006), and (iii) the quality of soil nutrients under these preferred trees probably affects the quality of nectar and pollen, and therefore explains the high activities of bees and consequently the high fruiting rates, yields and useful kernels in nuts (Chipojola 2009; Wunnachit 1991). The gene variability due to cross‐pollination delivered by bees might also explain the apparition of genetic material that enhances floral traits, bees' visitation and consequently cashew yields (Heard et al. 1990; O. M. Aliyu 2007).

Specifically, the high individual weight of raw nuts from non‐preferred cashew trees compared to the preferred trees might be attributed to the big shells that surround the kernel (A.C.I 2010). Probably, the genetic material from the non‐preferred trees might also affect the weight of shells, and therefore explain their high sizes of nuts (Chipojola 2009; Silué 2023).

## 5 Conclusion

Floral complex of study cashews is clearly differed with regard to their traits which encourage bee pollinators to establish recurrent visits and enhance pollen transferring and yields. The preferred cashew plants might contribute greatly to reducing the depletion of floral resources for bees, mainly during the dry season where there are few flowers in the north of Côte d'Ivoire. Future studies will examine whether these floral traits are also linked to genetic material from cashew plants, before the experimental breeding trials.

## Author Contributions

**Dolourou Silué:** conceptualization (equal), data curation (equal), formal analysis (equal), funding acquisition (equal), investigation (equal), methodology (equal), visualization (equal), writing – original draft (equal), writing – review and editing (equal). **Nicodénin A. Soro:** conceptualization (equal), methodology (equal), visualization (equal), writing – review and editing (equal). **Lombart M. M. Kouakou:** conceptualization (equal), methodology (equal), visualization (equal), writing – review and editing (equal). **Seydou Tiho:** conceptualization (equal), methodology (equal), visualization (equal), writing – review and editing (equal). **Souleymane Konate:** conceptualization (equal), methodology (equal), visualization (equal), writing – review and editing (equal). **Wouter Dekoninck:** conceptualization (equal), methodology (equal), validation (equal), writing – review and editing (equal).

## Funding

This work was supported by Royal Belgian Institute of Natural Sciences (CEBioS, GTI), (2023/SO1/ER1.1‐2/10), (2023/SO1/ER1.1/02). Laboratoire Mixe International (LMI), (LMI‐EDD/2022/01).

## Conflicts of Interest

The authors declare no conflicts of interest.

## Data Availability

Data that support the findings of this study are openly available in Figshare at: https://doi.org/10.6084/m9.figshare.28607690.v1.
